# Simulating Intravitreal Injections in Anatomically Accurate Models for Rabbit, Monkey, and Human Eyes

**DOI:** 10.1007/s11095-012-0721-9

**Published:** 2012-06-14

**Authors:** Paul J. Missel

**Affiliations:** Modeling and Simulation, Alcon Research Ltd., Mail Stop TC-47, 6201 South Freeway, Fort Worth, Texas 76134 USA

**Keywords:** Computational Fluid Dynamics (CFD), convective diffusion, drug clearance, intravitreal injection

## Abstract

**Purpose:**

To develop models for rabbit, monkey, and human that enable prediction of the clearance after intravitreal (IVT) injections in one species from experimental results obtained in another species.

**Methods:**

Anatomically accurate geometric models were constructed for rabbit, monkey, and human that enabled computational fluid dynamic simulation of clearance of an IVT injected bolus. Models were constructed with and without the retrozonular space of Petit. Literature data on clearance after IVT injection of substances spanning a range of molecular weight up to 157 kDa were used to validate the rabbit model.

**Results:**

The space of Petit had a significant increase on the clearance of slowly diffusing substances cleared by the anterior pathway by reducing the bottleneck for drug efflux. Models that did not include this zone could not accurately predict the clearance of slowly diffusing substances whose clearance was accelerated by intraocular pressure-driven convection.

**Conclusions:**

The ocular anatomy must be carefully reconstructed in the model to enable accurate predictions of clearance. This method offers an alternative means for scaling experimental data from one species to another that may be more appropriate than other simple approaches based entirely upon scaling of compartment volumes and flow rates.

## INTRODUCTION

Various types of numerical modeling that have been applied to the eye. Noncompartmental methods attempt to make predictions of drug exposure without using detailed models, and tend to focus entirely upon curve-fitting, requiring a large amount of experimental data. Classical compartmental pharmacokinetic modeling constructs a model consisting of compartments, typically assuming first-order transfer of material between compartments. The number of compartments and the equations describing transfer of material between them is derived empirically by the data (tissue concentration *versus* time). Physiologic-based pharmacokinetic (PBPK) models attempt to improve upon classical models by associating compartments with specific tissues and attempt to incorporate aspects of anatomy and physiology such as tissue volumes and fluid flow rates (1).

The modeling approach presented in this report uses the finite-volume method, an *in silico* method used in the engineering disciplines to model physical phenomena in particular systems. In this approach, an accurate geometric model is constructed, and the various regions are assigned physically meaningful material properties and boundary conditions, enabling the solution of equations describing the transport of fluid and drug by processes of diffusion and convection. As such it can be considered as an extension of PBPK compartmental modeling, as the explicit geometric models can associate compartments with particular tissues. Physiologic flows can be simulated numerically using flow boundary conditions, and some physiologic processes can be represented, for example convection within the aqueous humor driven by temperature differences between the cornea and iris (2,3).

In developing *in silico* models to enable accurate prediction of the distribution and clearance of intravitreally injected materials, various investigators have attempted to accurately represent the anatomy in the model geometry, and to incorporate as features to represent physiological properties and processes. Two previous models for the rabbit eye which deserve special consideration are those published by Friedrich and Park (4,5).

In our previous paper (6) an attempt was made to improve upon the geometry of the rabbit eye using insights that could be gained from imaging data available in the literature. One important anatomical feature that was incorporated in the geometry of the new model was the retrozonular space of Petit, a small gap between the anterior boundary of the vitreous and the ciliary body, which effectively extends the posterior portion of the aqueous humor nearly to the *ora serrata*, the anterior most portion of the retina. When simulating the clearance of intravitreally injected material, it was discovered that this very small feature exerts a rather important effect on the clearance behavior, and allows the model to much more accurately simulate ocular drug levels than the models of Friedrich and Park (6).

In this paper, the mechanism by which the space of Petit exerts its influence on the clearance of intravitreally injected materials was investigated. By conducting simulations of intravitreal injection in various geometric models of increasing complexity, it was possible to distinguish between effects that were of a purely geometric nature *versus* those that work through the influence of intraocular pressure. A simplified model for the eye was constructed comprised of a spherical vitreous, devoid of lens, bounded on one side with a shell representing the aqueous compartment. The interface to the aqueous compartment was constructed in one of two ways, including or excluding a small gap representing the canal. When the canal is included, the area of contact between the vitreous and aqueous compartments is expanded, enabling material diffusing toward the anterior chamber more ready access to clearance by the anterior pathway, reducing the bottleneck effect (6). The same strategy is used to develop anatomically accurate models for the human and cynomologous monkey eyes, incorporating the space of Petit and shaping anterior features as accurately as possible using literature imaging data for each species. The approach provides a new way to perform allometric scaling, the prediction of an experimental outcome in one species using data obtained from another.

## METHODS

### Software

Computational Fluid Dynamics (CFD) calculations were conducted using FLUENT software version 13.0.0 (ANSYS, Inc., Canonsburg, PA). Geometrical models were constructed using ANSYS DesignModeler version 13. Further preprocessing (meshing the geometry and creating entities to enable assignment of material properties and boundary conditions) for two-dimensional axisymmetric geometries was conducted using ANSYS Mesher version 13. For three-dimensional geometries, the geometry was exported from DesignModeler (Parasolid format) and imported into GAMBIT version 2.4.16 (also developed and distributed by ANSYS, Inc.). Simulations were conducted using a Dell Precision T7500 system with a Quad processor and 12 GB RAM running under Windows 7 Professional 64-bit.

### Geometry

**Fig. 1:**
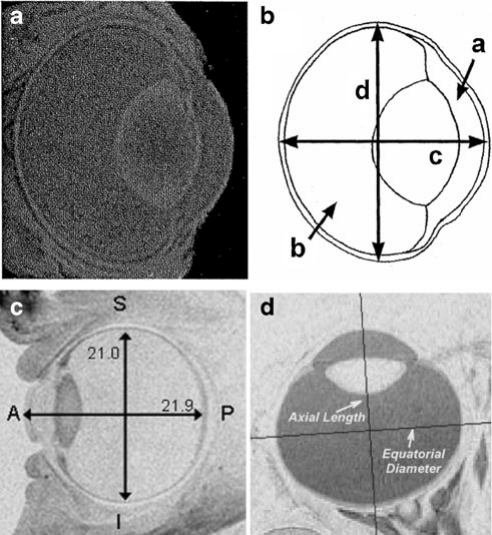
Representative high-resolution MRI images. (**a**, **b**) Image obtained from a rabbit eye, Fig. 4 of reference (7) (NZW rabbit, 1.5–2.7 kg). (**c**) Emmetropic human eye, Fig. 2c of reference (22). (**d**) 143-day old rhesus monkey eye, Fig. 7 of reference (29).

**Fig. 2:**
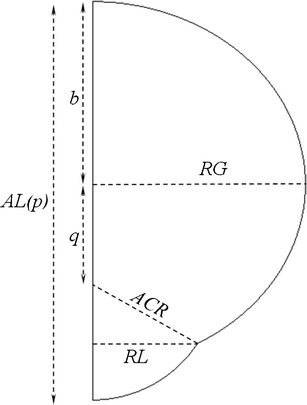
Outer surfaces of the ocular anatomy. Shown are the outer dimensions of the ocular models constructed in DesignModeler. Refer to Table I for descriptions.

A literature search was conducted to locate imaging data for constructing anatomically accurate models. Representative MRI images are shown in Fig. 1. Table I provides a description of the various dimensions used in the ocular models. The models are compared with characteristic dimensions of the literature models in Table II. Figure 2 illustrates the strategy for defining the outer surfaces of the geometry, using a circular arc to represent the outer corneal surface in the anterior portion and an ellipse for the remainder of the model. The definitions of these parameters are intended to conform to the norms adopted in the imaging literature for defining ocular anatomy. The posterior ellipse, centered at the origin, is defined by Eq. 1:

**Table I:** Key Dimensions of Ocular Models

*ACD*	Anterior Chamber Depth
*LT*	Lens Thickness
*VCD*	Vitreous Chamber Depth
*AL(O)*	Axial Length (Optical)
*AL(P)*	Axial Length (Physical)
*RV*	Radius Vitreous
*RG*	Radius Globe
*LR*	Lens Radius
*CBR*	Ciliary Body Radius
*IR*	Iris Radius
*RL*	Radius Limbus
*b*	Semi-major axis ellipse
*ACR*	Anterior Corneal Radius
*q*	Distance from ellipse origin to center of corneal arc

**Table II:** Comparison of Dimensions of Ocular Models (mm)

Item	Rabbit		Human		Cynomologous	
	Reference Value	Model Dimension	Reference Value	Model Dimension	Reference Value	Model Dimension
Anterior Cornea Curvature	–	8.29^a^	7.8 (19)	7.8	5.75 (28)	5.75
Posterior Cornea Curvature	–	8.01^a^	6.2–6.8 (19)	6.85	5.12 (28)	5.12
Cornea Thickness	0.33–0.38 (8)	0.36	0.52 (19)	0.52	0.4 (28)	0.4
Anterior Chamber Depth	2.34–2.49 (9)	2.45	3.28 (20) ^d^	3.27	3.24 (28)	3.24
	2.2 (10)					
Anterior Lens Curvature	–	5.76^a^	10 (19) 9.43^d^	7.31	10.34 (28)	9.35
Posterior Lens Curvature	–	4.79^a^	6 (19) 5.76^d^	5.3	6.39 (28)	4.3
Lens Thickness	6.7 (7)	6.7	3.89^d^	3.9	2.98 (28)	2.98
Lens Diameter	9.5 (7)	9.5	9.2 (21)	8.87	7.5 (21)	7.5
Inner CB Diameter		14.0^a^	11.0 (21)	11.0	8.9 (21)	8.94
Axial Length (Optical)	16.3–16.6 (9)	15.37^c^	23 (22)	22.065	17.92 (28)	17.12^k^
Axial Length (Physical, AL + r/c/s)	16 (7)	16.05	23.205^g^	23.205	18.92^g^	18.12^k^
Diameter vitreous cavity		16.74	22.55 (22)	22.55	18.24^i^	17.39^k^
Diameter globe (Dvit + 2*r/c/s)	18 (7)	18.0	24.83^h^	24.83	20.24^h^	19.39^k^
Retinal Thickness	0.11 (11,12)	0.1	0.21–0.25 (23)	0.22	0.2^j^	0.2
Choroidal Thickness		0.2	0.32 (24)	0.32	0.4^j^	0.4
Scleral Thickness	0.2–0.5 (13)	0.33	0.6 (25)	0.6	0.4 (31)	0.4
Diameter Limbus	13.74 (10)	13.74	11.77 (10)	11.75	9.6 (32)	9.6
Vitreous chamber depth		6.27		14.89		10.90
Pupil Diameter	6 (14,15)^b^	6	3.9 (3)	4	4.9 (33) 3.1 (34)	4
Aq. Hum. production *f* (μL/min)	3.0 (16,17)	3.0	2.5^e^	2.5	1.71 (33) 2.7 (35)	1.7
Volume vitreous V_v_ (mL)	1.7 (18)	1.52^c^	4.7^f^; 5 (18)	4.85	2.05 (36) 1.71 (37)	2.17
Volume aqueous humor (mL)		0.325^c^		0.238		0.134

^a^ Estimated from MRI image of rabbit eye in Fig. 2 of reference (7). Corneal and lens curvatures adjusted as necessary to match boundaries determined from image

^b^ Average of several measurements from various figures of Ceckler 1991 assuming lens diameter was 9.5 mm. Also the baseline pupil diameter in Ogidigben 2001 was 6 mm

^c^ Reduced slightly from reference value to achieve target rabbit vitreous volume of 1.52 mL to match population average from recent study using mixed breed rabbits

^c^ Model adjusted to match population average volumes for vitreous and aqueous compartments from recent studies using mixed breed rabbits

^d^ Calculated from regression values for MRI measurements of reference (20)

^e^ Average of all the values from Table I of reference (26) for numerous studies involving normal subjects

^f^ Average total vitreous volume from reference (27) for age range 25–75 years

^g^ Added thicknesses of retina, choroid and sclera to optical axial length

^h^ Added twice the thicknesses of retina, choroid and sclera to diameter of the vitreous cavity

^i^ Calculated to match the human ratio of globe diameter/physical axial length =1.07, which is also very close to the ratio measured from infant rhesus monkeys in reference (29)

^j^ Attributed one third the thickness of the choroid-retinal layer for untreated eyes from reference (30) to the retina and two thirds to the choroid

^k^ Reduced slightly to achieve target cyno vitreous volume of 2.17 mL to match population average from recent study

1 $$ \frac{{{x^2}}}{{R{G^2}}} + \frac{{{y^2}}}{{{b^2}}} = 1 $$

From the diagram of Fig. 2, the semi-minor axis of the ellipse *b* is defined in terms of the other dimensions by Eq. 2:

2 $$ b = AL(p) - q - ACR $$

The equation for the circle defining the anterior corneal surface is given by Eq. 3:

3 $$ {x^2} + {\left( {y + q} \right)^2} = AC{R^2} $$

The value of *q*, the distance from the origin of the posterior ellipse to the center of corneal arc, is obtained by finding the simultaneous solution to Eqs. 3 and 4, where *RL*, the limbus radius is substituted for *x* and the expression for *b* from Eq. 2 was substituted in Eq. 1 to provide Eq. 4. The expression for *q* is given by Eq. 5.

4 $$ \frac{{R{L^2}}}{{R{G^2}}} + \frac{{{y^2}}}{{{{\left( {AL(p) - q - ACR} \right)}^2}}} = 1 $$

5 $$ q = \frac{{\left( \begin{gathered} RG\sqrt {{\left( {R{G^2} - R{L^2}} \right)\left( {2\left( {AL(p) - ACR} \right)\left( {\sqrt {{AC{R^2} - R{L^2}}} - ACR} \right) - R{L^2} + AL{{(p)}^2}} \right)}} \hfill \\ \quad \quad \quad \quad \quad \quad + \left( {ACR - AL(p)} \right)\left( {R{G^2} - R{L^2}} \right) - R{G^2}\sqrt {{AC{R^2} - R{L^2}}} \hfill \\ \end{gathered} \right)}}{{R{L^2}}} $$

**Fig. 3:**
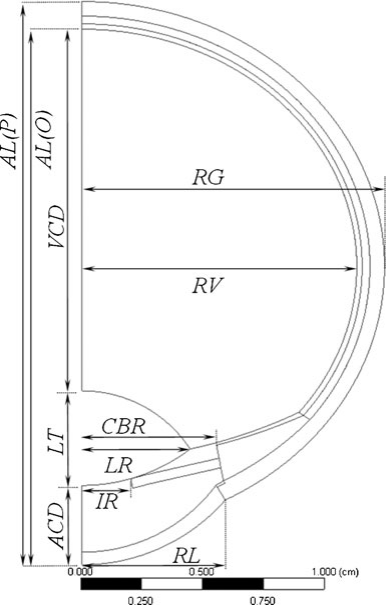
Interior structure of the human ocular anatomy. See Table I for descriptions of the various dimension labels.

**Fig. 4:**
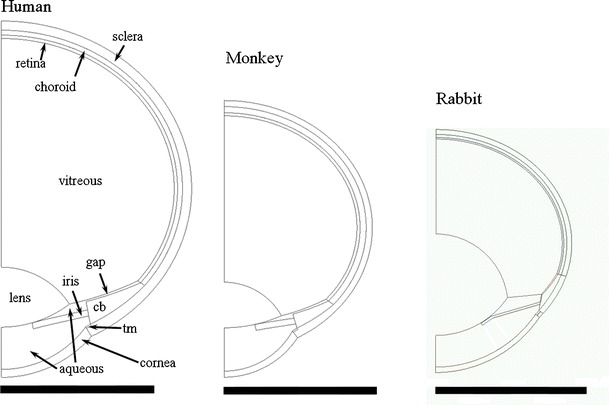
Comparison of ocular models. cb, ciliary body; tm, trabecular meshwork; gap, retrozonular space of Petit.

Figure 3 shows interior ocular structures for the human ocular anatomy with dimension labels from Table I. The geometries for all three species were constructed similarly. Key radii and coordinates for the models for each species are given in Tables III (rabbit), IV (human) and V (monkey). The geometric profiles for each of the models are compared on the same size scale in Fig. 4, which also includes labels identifying the various tissue regions applied to the human geometry.

**Table III:** Key Radii and Coordinates (cm) for the Rabbit Eye Model

	R1	R2	X-cent	Z-cent
Outer sclera	0.900	0.753	0	0
Choroid-sclera boundary	0.867	0.720	0	0
Retina-choroid boundary	0.847	0.700	0	0
Vitreous-retina boundary	0.837	0.690	0	0
Lens rear	0.479	0.479	0	−0.415
Lens front	0.576	0.576	0	−0.031
Cornea outside	0.829	0.829	0	−0.023
Cornea inside	0.801	0.801	0	−0.015
Curved portion of hyaloid	0.941	0.941	0	0.295
Curved cb adjacent to hyaloid	0.965	0.965	0	0.321
Curved cb adjacent to sclera	0.904	0.904	0	0.149
Intersection lens equator with hyaloid	–	–	0.475	−0.357
Intersection curved & flat hyaloid	–	–	0.700	−0.335
Intersection curved hyaloid & retina	–	–	0.801	−0.200
Intersection outer sclera, outer cornea	–	–	0.687	−0.487
Intersection inner sclera, inner cornea	–	–	0.661	−0.468
Intersection cb, tm, aqueous, sclera	–	–	0.684	−0.442

– Not Applicable

**Table IV:** Key Radii and Coordinates (cm) for the Human Eye Model

	R1	R2	X-cent	Z-cent
Outer sclera	1.2415	1.0918	0	0
Choroid-sclera boundary	1.1815	1.0318	0	0
Retina-choroid boundary	1.1495	0.9998	0	0
Vitreous-retina boundary	1.1275	0.9778	0	0
Lens rear	0.53	0.53	0	−1.041
Lens front	0.731	0.731	0	−0.170
Cornea outside	0.78	0.78	0	−0.449
Cornea inside	0.685	0.685	0	−0.492
Curved portion of hyaloid	2.091	2.091	0	1.292
Curved cb adjacent to hyaloid	2.251	2.251	0	1.448
Curved cb adjacent to sclera	1.327	1.327	0	0.311
Intersection lens equator with hyaloid	–	–	0.444	−0.751
Intersection curved hyaloid & retina	–	–	0.890	−0.6
Intersection outer sclera, outer cornea	–	–	0.587	−0.962
Intersection inner sclera, inner cornea	–	–	0.550	−0.900
Intersection cb, tm, aqueous, sclera	–	–	0.580	−0.882

– Not Applicable

**Table V:** Key radii and coordinates (cm) for the monkey eye model

	R1	R2	X-cent	Z-cent
Outer sclera	0.970	0.831	0	0
Choroid-sclera boundary	0.930	0.791	0	0
Retina-choroid boundary	0.890	0.751	0	0
Vitreous-retina boundary	0.870	0.731	0	0
Lens rear	0.43	0.43	0	−0.789
Lens front	0.935	0.935	0	0.278
Cornea outside	0.575	0.575	0	−0.406
Cornea inside	0.512	0.512	0	−0.429
Curved portion of hyaloid	1.502	1.502	0	0.876
Curved cb adjacent to hyaloid	1.644	1.644	0	1.014
Curved cb adjacent to sclera	1.114	1.114	0	0.349
Intersection lens equator with hyaloid	–	–	0.375	−0.578
Intersection curved hyaloid & retina	–	–	0.666	−0.47
Intersection outer sclera, outer cornea	–	–	0.480	−0.722
Intersection inner sclera, inner cornea	–	–	0.447	−0.678
Intersection cb, tm, aqueous, sclera	–	–	0.476	−0.658

– Not Applicable

The Canal of Petit is defined as the space between the posterior and hyaloid zonules, which extend outward to the ora serrata at the anteriormost boundary of the retina (see Figure 12.43 of reference (19)). Although the canal could not be resolved in MRI images, its location was inferred from the overall shape of the ciliary body and the location of the pars plana. The thickness of the canal was set to 0.1 cm for all species.

**Fig. 5:**
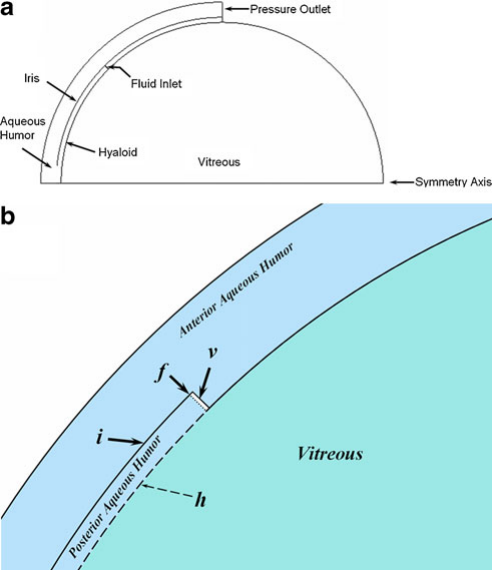
Simplified ocular model of a spherical vitreous with partial aqueous shell. This model does not contain the gap representing the retrozonular space of Petit. (**a**) Entire 2-D geometry, which when rotated about the horizontal axis produces the model in three dimensions. (**b**) Close-up showing features near the fluid inlet (dotted line at ***f***): ***i***, iris; ***v***, void to allow separation of fluid inlet from the aqueous humor domain; dashed line ***h*** is the hyaloid boundary between the non-porous aqueous and the porous medium of the vitreous. Material transport (fluid and/or drug) is allowed across the hyaloid; solid lines are barriers to transport.

**Fig. 6:**
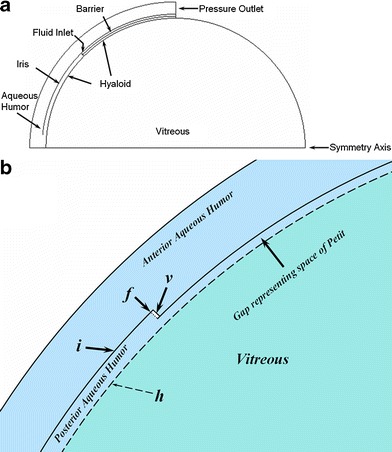
Simplified ocular model of a spherical vitreous with partial aqueous shell. This model contains the gap representing the retrozonular space of Petit. (**a**) Entire 2-D geometry, which when rotated about the horizontal axis produces the model in three dimensions. (**b**) Close-up showing features near the fluid inlet (similar features as in Fig. 5b). This model inserts a gap representing the space of Petit which expands the area of contact between aqueous humor and the vitreous medium, increasing the opportunity of transport between these compartments.

Additionally, two simplified models (Figs. 5 and 6) were constructed in order to gain insight into the way in which aspects of the ocular anatomy influence the elimination of intravitreally injected materials from the eye. In each model the vitreous is represented by a semicircle, which when rotated about the symmetry axis becomes a simple sphere of volume 1.52 mL. The aqueous humor compartment is a partial shell which extends from the symmetry axis to a point halfway along the circular arc of the vitreous. The thickness of the shell is chosen to provide a volume of 0.325 mL for the total aqueous compartment. The interface between vitreous and aqueous compartments was constructed in one of two ways, either with or without a thin gap separating the vitreous compartment from the aqueous humor.

In the model without the gap (Fig. 5), the contact between these compartments is along the hyaloid boundary as shown, in this case extending to an angle of 45° from the symmetry axis. A fluid inlet is placed at the beginning of the point of contact, extending radially outward to the beginning of the iris. The iris follows a curved trajectory at a constant radius from the center of the semicircle, and extends from the fluid inlet towards the symmetry axis and stops at a point shortly above the symmetry axis. The point where the iris boundary stops represents the pupil opening. The iris constitutes a wall boundary to flow of both fluid and drug. Another flow boundary separates the aqueous and vitreous compartments along the remainder of the aqueous shell on the other side of the flow inlet (angle greater than 45°). Some details are more easily seen in the close-up of Fig. 5b. A very small empty space was inserted in the aqueous humor compartment to allow for the separation of the flow inlet.

The second simplified model including the gap is depicted in Fig. 6. It is similar in overall construction to the previous model, but the gap allows for a much larger region of contact between the two compartments. This second model retains the same positions for the iris and for the hyaloid boundary extending out to the fluid inlet. But in this second model, the flow boundary that extended past the fluid inlet has been pushed out radially to allow the formation of a gap. The hyaloid boundary now extends along the entire separation between the aqueous and vitreous compartments. This is more clearly seen in the close-up of Fig. 6b. This gap represents the retrozonular space of Petit. The size of the empty space allowing for the separation of the fluid inlet has been reduced accordingly, as has the width of the fluid inlet.

### Theory: Rate of Egress of Intravitreally Injected Substances

For some intravitreally injected substances, the only means of egress is by diffusion from the vitreous and through the hyaloid boundary separating the vitreous and aqueous compartments. Once the material enters the aqueous humor compartment it is eliminated by the natural production of aqueous humor at the ciliary body and drainage through the trabecular meshwork into the canal of Schlemm. A very simple expression can be derived for the ratio of drug concentration in the aqueous humor divided by the drug concentration in the vitreous once the system reaches quasi-steady state, assuming that the aqueous compartment is well-stirred (18). The amount of drug lost from the vitreous per unit time is:

6 $$ Vitreous\;Loss\;Rate = {k_f}\;{C_v}\;{V_v} $$

where *k*_*f*_ is the fraction of material lost from the vitreous per unit time, *C*_*v*_ is the average vitreous concentration, and *V*_*v*_ is the volume of the vitreous compartment. The amount of drug lost from the aqueous per unit time is:

7 $$ Aqueous\;Loss\;Rate = f\;{C_a} $$

where *f* is the aqueous humor flow rate (3 μL/minute for the rabbit) and *C*_*a*_ is the average concentration of drug in the aqueous compartment. At quasi-steady-state these two expressions can be equated:

8 $$ {k_f}\;{C_v}\;{V_v} = f\;{C_a} $$

Rearranging, this relationship can be expressed as follows:

9 $$ \frac{{{C_a}}}{{{C_v}}} = \frac{{{k_f}{V_v}}}{f} $$

### Simulation of Drug Advection in the Eye Using Fluent

Fluent software enables the simultaneous solution of equations describing convection and diffusion. In the absence of source terms the convective diffusion equation is solved in three dimensions (38):

10 $$ \frac{{dC}}{{dt}} + \overrightarrow v \cdot \nabla C = D{\nabla^2}C $$

where *C* is concentration, *t* is time, *D* is the diffusion coefficient and $\overrightarrow v$is the velocity vector. The aqueous humor is considered to be a simple Newtonian fluid with the properties of water, and thus the Navier–Stokes equations are solved to obtain the fluid velocity in this domain (39):

11 $$ \rho \frac{{d\overrightarrow v }}{{dt}} = - \nabla p + \eta \;{\nabla^2}\overrightarrow v + \overrightarrow F $$

where *ρ* is the fluid density, *p* is pressure, *η* is fluid viscosity, and the term $\overrightarrow F$ refers to body forces (such as gravity) which are neglected. All other regions are considered to be porous media. Using Darcy’s Law, the local volume flux rate for fluid creeping through a porous medium is related to the local pressure gradient by Eq. 12 (40):

12 $$ \overrightarrow v = - \frac{{{K_h}}}{\eta }grad(p) $$

where *K*_*h*_ is the permeability of the porous medium to fluid flow. Assuming conservation of matter (39), $div\left( {\overrightarrow v } \right) = 0$, thus:

13 $$ div\left( {\frac{{{K_h}}}{\eta }\;grad(p)} \right) = 0 $$

### Material Properties and Boundary Conditions

To simulate the distribution and clearance of an intravitreally injected material, the model must account for the following potential mechanisms for drug transport and egress:

- Radial outward diffusion with potential clearance by the choroidal vasculature
- Diffusion toward the front of the eye with clearance by the aqueous humor turnover
- Hydraulic clearance by pressure-assisted convection through the outer sclera

These factors are accounted for by means of assigning appropriate material properties and boundary conditions. In the following sections are discussed the fluid region type and tissue hydraulic properties, material diffusivity in the various tissues, and boundary conditions.

Whenever possible it is prudent to anchor selected model parameters by referencing *in vitro* or *ex vivo* experiments in which these values are determined independently. In this way the model is not burdened to attempt to determine each parameter by fitting the combination of values that would provide the best fit of simulation results to experimental data. This is possible for some measurements of hydraulic resistivity and diffusivity as described below.

#### Fluid Region Type/Tissue Hydraulic Resistivity

The aqueous humor is the only non-porous fluid region; all other regions are porous media. The viscosity of fluid in each region type was 6.9 × 10^−4^ kg/m-s, the value appropriate for water at 37°C.

The software solves for the appropriate model of fluid flow for each region according to its assignment type (Navier–Stokes flow for a simple fluid, Darcy Law flow for porous media), in a manner that is transparent to the user. The hydraulic resistances assigned to various tissue regions are shown in Table VI. The value for the vitreous was taken from reference (41). The last value in the table corresponds to the value for sclera taken from reference (42). (These values are about 43% higher than those used in the previous study (6), which was conducted using the value of viscosity for water at 20°C. The porous medium flow solution is equivalent between this study and the previous study, since the values of hydraulic resistance scale with the value of fluid velocity assigned to the percolating fluid.) The values hydraulic resistance applied to the trabecular meshwork for the various species were adjusted to provide an intraocular pressure of 10.1, 15 and 20 Torr (mm Hg), and probably do not reflect meaningful species differences amongst the hydraulic resistances of the trabecular meshwork.

**Table VI:** Hydraulic Resistance Values Assigned to Various Tissue Regions

Tissue	Hydraulic Resistivity (M^−2^)
Vitreous	*1.725 × 10^13^
Trabecular Meshwork, human	**0.01–2.88–6.03 × 10^15^
Trabecular Meshwork, rabbit	**0.06–2.94–6.10 × 10^15^
Trabecular Meshwork, monkey	**0.04–3.51–7.35 × 10^15^
All other tissues except aqueous humor	*9.66 × 10^17^

*Values derived from experimental measurements of references (41,42) for a temperature of 37°C.

**The three values correspond to the values required to obtain maximum intraocular pressures of 10.1, 15 and 20 Torr within each of the species models as indicated.

#### Diffusivity in Various Tissue Regions

Table VII presents literature data on measurements of diffusivity of some materials which have been injected intravitreally in rabbit. These diffusivities were measured in aqueous media (buffer or water) at either 20 or 25°C. To calculate the diffusivity expected in aqueous media at 37°C, Eq. 14 was used (43).

14 $$ \frac{{{D_2}}}{{{D_1}}} = \left( {\frac{{{T_2}{\eta_1}}}{{{T_1}{\eta_2}}}} \right){\left( {\frac{{M{W_1}}}{{M{W_2}}}} \right)^{{1/3}}} $$

where the subscript 1 refers to the material for which the diffusivity is known (at the measurement temperature *T*_1_), subscript 2 denotes the material for which the diffusivity is desired to be calculated (at the temperature *T*_2_), *MW*_1,2_ denotes the molecular weights of the two materials and *η*_1,2_ denotes the viscosity of aqueous medium at the two temperatures. In the present case the values of viscosity for pure water at the various temperatures were used.

**Table VII:** Literature Reference Measurements of Material Diffusivities in Aqueous

Material	Molecular Weight	Measured Diffusivity (cm^2^ s^−1^)	Diffusivity calculated for 37°C (cm^2^ s^−1^)
Fluorescein	332	5.5 × 10^−6^ (20°C)^a^	8.43 × 10^−6^
		6.4 × 10^−6^ (25°C)^b^	8.57 × 10^−6^
Sucrose	342.3	5.25 × 10^−6^ (25°C)^c^	7.03 × 10^−6^
Bovine Albumin	68,000	6.8 × 10^−7^ (20°C)^d^	1.04 × 10^−6^

^a^Ref. (44), ^b^Ref. (45), ^c^Ref. (46), ^d^Ref. (47)

Table VIII shows the values of diffusivity that were used in the simulations in the present study. The values for diffusivity for all the materials in the vitreous and aqueous compartments were considered to be equivalent to the diffusivity of the materials in pure water at 37°C. The diffusivities for the dextran polymers were calculated using Eq. 15:

**Table VIII:** Diffusivities (cm^2^ s^−1^) of Materials Applied in Various Tissue Regions

Material	Molecular Weight	Diffusivity in Vitreous, Aqueous	Diffusivity in Cornea, Sclera	Diffusivity in Retina, Choroid, Iris, Ciliary Body
Fluorescein	332	8.5 × 10^−6^	6.375 × 10^−7^	6.375 × 10^−8^
Sucrose	342	7 × 10^−6^	5.25 × 10^−7^	5.25 × 10^−8^
Bovine Albumin	68,000	1.04 × 10^−6^	7.8 × 10^−8^	7.8 × 10^−9^
Dextran D10	10,500	1.62 × 10^−6^	1.215 × 10^−7^	1.215 × 10^−8^
Dextran D67	67,000	6.06 × 10^−7^	4.545 × 10^−8^	4.545 × 10^−9^
Dextran D157	157,000	3.86 × 10^−7^	2.895 × 10^−8^	2.895 × 10^−9^

15 $$ D = \frac{{{k_B}T}}{{6\pi \eta {r_H}}} $$

where *k*_*B*_ is the Boltzmann constant and *r*_*H*_ is the hydrodynamic radius of the polymer calculated from the empirical relationship of Eq. 16 (48).

16 $$ {r_H} = 0.015\;M{W^{{0.53}}} $$

Note that at the molecules become very large there is the possibility that the diffusivity in the vitreous may slow down because of entanglement or interaction with the biopolymeric components of vitreal fluid. In bovine vitreous, the diffusivity of Dextran D157, for example, would be expected to be reduced by nearly 20% (49). However, it will be demonstrated that the vitreous diffusivities for the Dextran polymers presented in Table VIII enable the simulations of the current study to provide a reasonable fit to the experimental data for the rabbit vitreous. Thus no attempt was made to reduce the vitreous diffusivities for the larger molecules.

The diffusivities assigned to material in the cornea and sclera were 7.5% of the value assigned in the vitreous/aqueous. The diffusivities assigned in the retina, choroid, iris and ciliary body were 0.75% of the value assigned in the vitreous/aqueous. These are the same ratios of diffusivity in the various tissues deduced in studies of the partitioning and transport of AL-4940, an active metabolite of Anecortave Acetate in the rabbit eye (50). These ratios correspond to diffusivities in the cornea and retina that are about 1/13^th^ and to diffusivities in the remaining tissues that are about 1/130^th^ the value in the vitreous and aqueous. The ratio of 1/13 is about a factor of two-fold off from the ratio of about 1/6^th^ that was measured for a variety of small (51) and large (52) molecules.

Ideally it would be desirable to independently measure the diffusivities of each of these materials in each of the various tissue compartments in order to enable accurate estimation of material concentrations in these tissues. However for our current purposes we are interested mainly in simulating the egress of drug from the vitreous compartment, and thus this “one-size-fits-all” approach to maintaining a constant ratio of tissue diffusivities adequate for this study.

The lens was absent in all simulations except for one case, in which the lens was included as a porous medium. The hydraulic resistance and diffusivity assigned to the lens were the same as for the sclera. The purpose of this simulation was not to attempt to accurately simulate hydraulic flow or material advection in the lens, but rather to qualitatively investigate the potential impact of the lens on the clearance of intravitreally injected materials.

#### Boundary Conditions for Mass Flow and Pressure

**Fig. 7:**
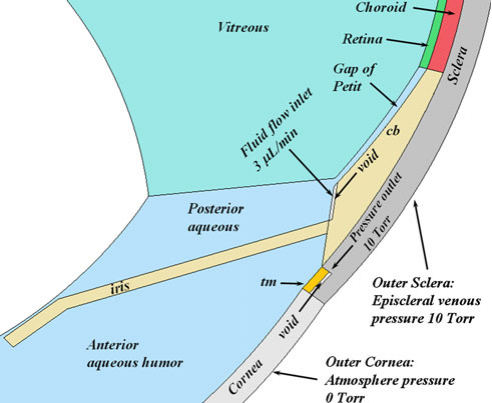
Close-up of the rabbit model geometry showing the position of the fluid inlet and the pressure outlet. cb, ciliary body; tm, trabecular meshwork. For 2-D axisymmetric geometries, a void was placed behind the fluid inlet and pressure outlet as shown. The void was not required for 3-D geometries since surfaces could be split. The outer surfaces of the cornea and sclera were also defined as pressure outlets with the boundary pressure conditions shown.

A fluid inlet boundary condition was imposed on the curved surface of the ciliary body (cb) in direct contact with the posterior aqueous humor. A pressure outlet was imposed at the rear of the trabecular meshwork (tm), a thin annular portion of the sclera just outside the cornea. The placement of the fluid inlet and pressure outlet in the axisymmetric model for the rabbit eye are shown in Fig. 7. The axisymmetric models required insertion of a very small gap behind the inlet and outlet to enable these surfaces to become exterior walls, enabling the boundary conditions to be applied correctly. In the full 3-dimensional models the voids were not required as it was possible to split the surfaces and to define one surface as an impenetrable wall, so that boundary conditions could be applied to the other wall resulting from the split.

These two boundary conditions permitted the orderly flow of fluid from the ciliary body around the iris and out through the trabecular meshwork. The aqueous humor production rates used for the various species were taken from the literature and appear in Table II. Also shown in Fig. 7 are the pressure boundary conditions for the exterior cornea and exterior sclera, which were also defined as pressure outlets. The gauge setting for the pressure outlet behind the trabecular meshwork and for the outer sclera was 10 Torr, corresponding to the episcleral venous pressure which is about 9 or 10 Torr (53–55).

In simulations in which only the vitreous and aqueous compartments were represented, the fluid inlet was at the same location but the pressure outlet was moved to the edge corresponding to the front surface of the trabecular meshwork (which was also not present in the model for these simulations).

All other boundaries between various tissue compartments were set to type porous-jump with default settings, which assigns a zero value for the boundary thickness, so that the boundary itself does not impose a pressure drop.

#### Boundary Conditions for Concentration

Diffusant concentration was represented in Fluent by a user-defined scalar (uds-0). The flux boundary condition on surfaces is expressed according to Eq. 17:

17 $$ {\left. { - D\frac{{\partial C}}{{\partial r}}} \right|_{{surface}}} = {\left. { - P * C} \right|_{{surface}}} $$

The default condition applied to exterior wall surfaces in Fluent is the zero flux condition, which is equivalent to setting the value of *P* to zero. This condition ensures that there is no production or elimination of uds-0 along the boundary. It is also possible to assign a value condition on a wall surface. A zero value condition was applied to the fluid inlet for all simulations.

When simulating intravitreal injection of Fluorescein, a user-defined function was programmed to assign a flux condition for non-zero values of *P* on the outer vitreous boundary, and the value of concentration was set to zero on the boundaries between the aqueous humor and the ciliary body and iris. For all other simulations, the default zero flux condition was used for all surfaces, and thus the only means by which drug can leave the system is by diffusion toward and across the hyaloid membrane where it is subsequently caught up in the natural process of aqueous humor convection and elimination through the pressure outlet. It is also possible to enable or disable accounting for diffusion of the user-defined scalar through the fluid flow inlet. Except were indicated differently, in this study accounting for diffusion through the inlet was disabled.

#### Initial Condition

The initial condition was to set the value of the user-defined scalar to a value representing the concentration of injected material within a 10 or 20 μl sphere and zero elsewhere. In most cases the bolus was placed on the symmetry axis about halfway between the lens and the rear of the vitreous. In one simulation in the 3-D model for the rabbit eye, the bolus was placed away from the symmetry axis on the side of the vitreous.

#### General Solution Procedure

First, the steady-state flow solution was obtained by enabling only the equations for pressure and momentum. For simulations in which all tissue compartments were represented, the hydraulic resistance of the trabecular meshwork was adjusted until the desire intraocular pressure was obtained. In solutions in which only the vitreous and aqueous compartments were represented, no adjustment of hydraulic resistance was necessary. Once the flow solution was obtained, the pressure and velocities were frozen, the pressure and velocity equations were disabled, the equation for the user-defined scalar was enabled, and the time-dependent solution for the advection of the user-defined scalar in the established flow field was obtained using 600 s time steps.

## RESULTS

The various types of models examined and location of various results are summarized in Table IX.

**Table IX:** Summary of Model Types and Results

Description	Geometry	Flow Analysis	Transport Analysis
Anatomical model, aqueous and vitreous only, no space of Petit	Figures 4 and 7 (subset)	Figures 8, 9 and 10	Figure 18
Anatomical model, all tissues, including space of Petit	Figures 4 and 7	Figures 11, 12	Figures 14b, c, 16, 18, 19
Simplified axisymmetric model without gap	Figure 5	Figure 13	Figures 14d, 17
Simplified axisymmetric model including gap	Figure 6	N/A	Figure 17

### Flow Solution

**Fig. 8:**
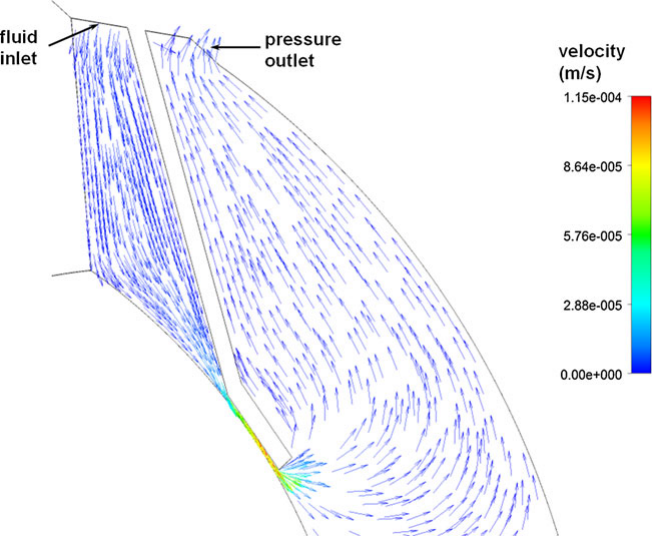
Vector velocity plot showing flow of aqueous humor in the region near the iris. Vectors have equal length and are color coded by velocity.

**Fig. 9:**
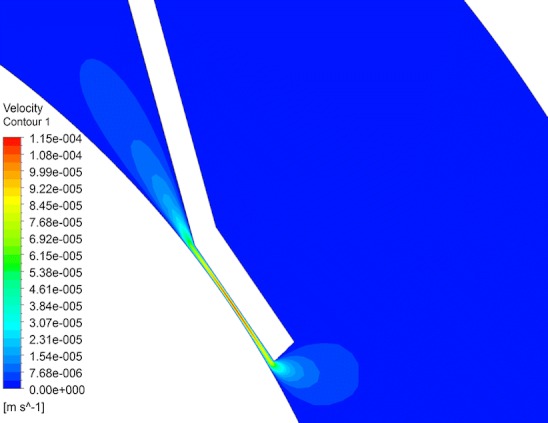
Contour plot of fluid velocity magnitude in the aqueous humor near the iris.

**Fig. 10:**
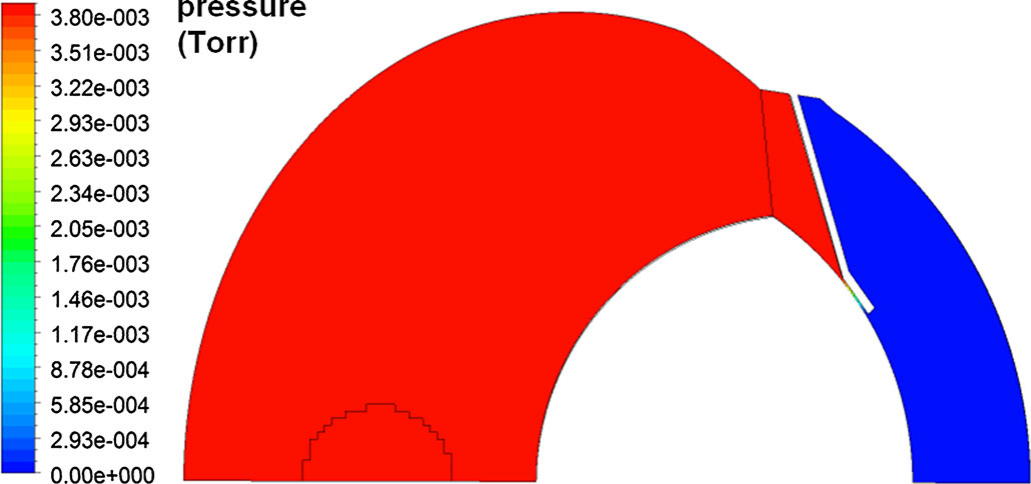
Contours of pressure in rabbit ocular model comprised only of the vitreous and aqueous humor without the gap.

**Fig. 11:**
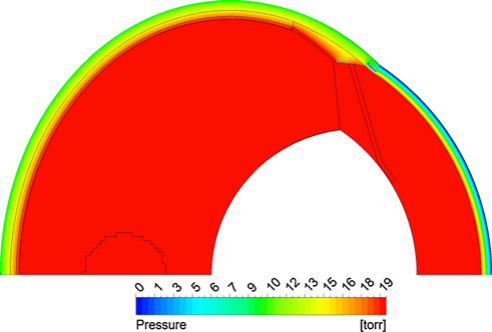
Contours of pressure in rabbit model including all tissue layers for the case in which the maximum intraocular pressure was adjusted to 20 Torr.

**Fig. 12:**
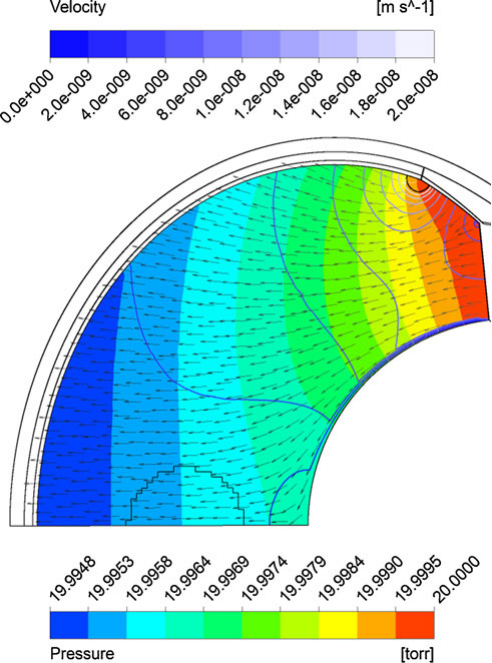
Superposition of velocity and pressure in the vitreous. *Solid colors* show pressure. *Line contours* show fluid velocity magnitude. Arrows show direction of fluid flow.

Figure 8 shows fluid velocity vectors for aqueous humor in the region near the iris for the rabbit model comprised only of the vitreous and aqueous compartments of the anatomically accurate model of the rabbit eye without the space of Petit. This orderly pattern of fluid flow is virtually the same when the gap is added, and when all the tissues are included in the model. Figure 9 shows contours of fluid velocity. The maximum velocity of about 1 × 10^−4^ m/s occurs in the region of closest approach between the iris and the lens. Figure 10 shows contours for pressure in the model. The maximum pressure is about 4 millitorr, with the entire pressure drop occurring in the region of closest approach of the iris to the lens, which accounts for most of the resistance to flow. Figure 11 shows the pressure distribution in the rabbit model when all tissues are present and the trabecular meshwork hydraulic resistivity is adjusted to achieve a maximum intraocular pressure of 20 Torr. The entire ocular interior is at virtually the same pressure, with the entire pressure drop occurring in the outer shell tissues. To understand how pressure drives convective flow in the vitreous, in Fig. 12 a contour plot of the pressure distribution in the vitreous is shown, scaled to the range of pressure established within the vitreous. Also shown in Fig. 12 is an overlay of contours of velocity magnitude. The pressure drop across the entire rabbit vitreous is only about 5 millitorr, but this is sufficient to produce a percolating fluid flow with a maximum velocity of about 3 × 10^−8^ m/s. While this seems like a very slow flow, as will be seen it is sufficient to exert an influence on the convective diffusion of large molecules in the vitreous. The velocity vectors visualize the flow direction which can be seen to follow the direction of the pressure gradient. The pressure and flow solutions in the other species were quite similar in all respects.

**Fig. 13:**
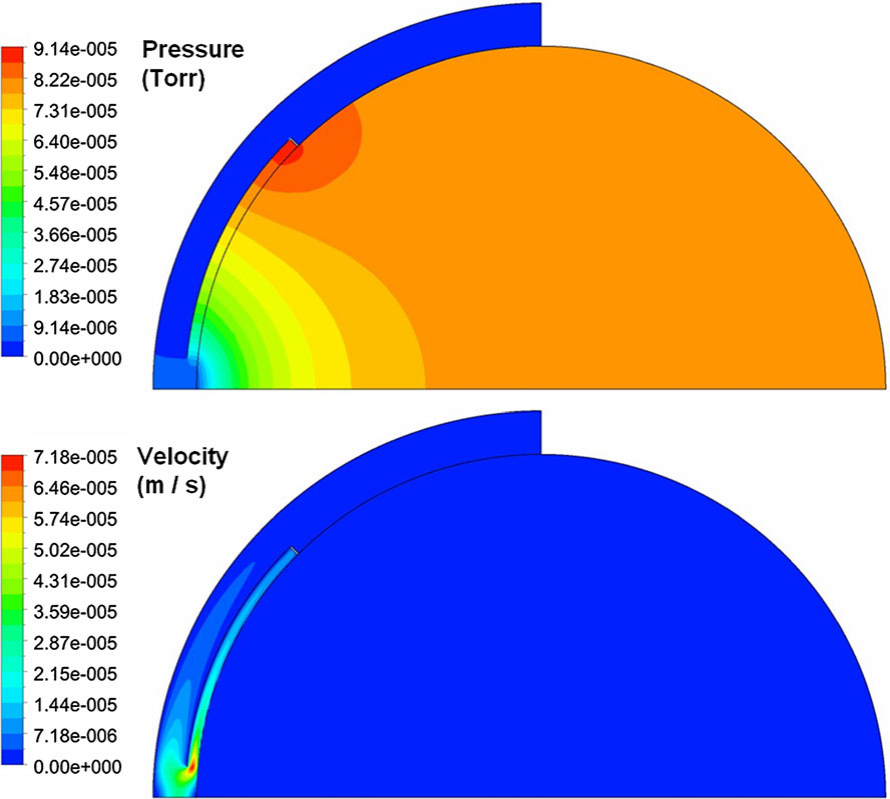
Contours of pressure and fluid velocity magnitude for the simplified ocular model without the gap.

Figure 13 shows the pressure and velocity magnitude plots for the simplified ocular model without the gap. The maximum pressure is about 0.1 millitorr, and the fluid velocity in the aqueous humor achieves a maximum value on the order of 10^−4^ m/s, comparable to the maximum velocity in the aqueous humor of the anatomically accurate rabbit model.

### Solution for Concentration of Injected Material

**Fig. 14:**
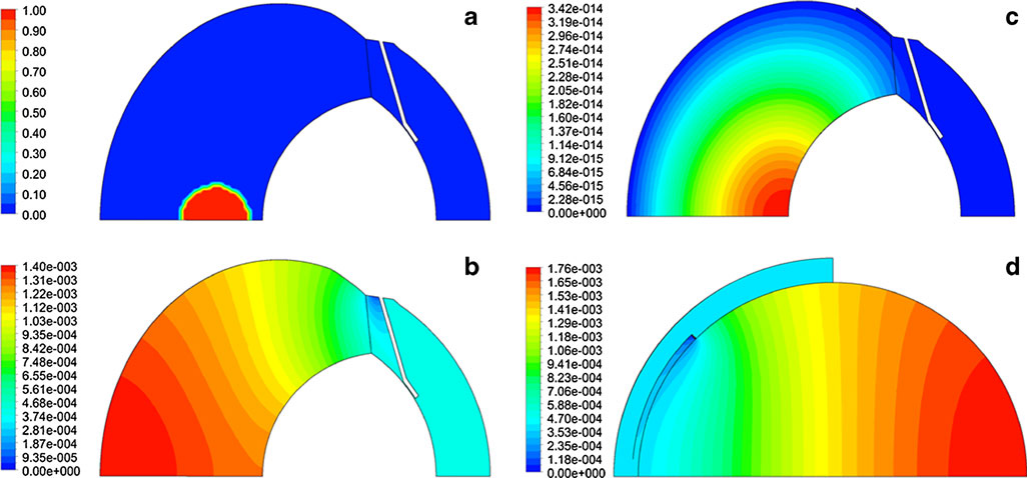
Contours of concentration. (**a**) Initial location of bolus. (**b**) Concentration contours 48 hours after injection of sucrose. (**c**) Contours 48 hours after injection of fluorescein when an infinite sink was applied on all surfaces of the iris, ciliary body, and exterior vitreous. (**d**) Contours for sucrose 48 hours after injection in the simplified axisymmetric model, 45° opening without the gap.

Figure 14 shows representative concentration plots in the rabbit model. The first panel shows the initial condition for concentration, a spherical bolus on the symmetry axis. Also compared are the concentration contours 48 h after injection of sucrose or fluorescein, sufficient time to achieve the quasi steady-state; at later times the contours appear the same and the maximum concentration decreases exponentially with time. The contours are quite different for these two molecules, because sucrose can only leave the vitreous by the anterior pathway, whereas fluorescein can be eliminated through the exterior vitreous. For sucrose, the concentration contours are perpendicular to the outer vitreous boundary for both the anatomically accurate and simplified models, with the highest concentration remaining at the rear of the vitreous. At the end of 48 h, the maximum concentration values are also quite different, being about 3.4 × 10^−14^ for fluorescein and 1.4 × 10^−3^ for sucrose in the anatomically accurate model (expressed in units of concentration of the injected bolus). The sucrose concentration at 48 h in the simplified model is 1.8 × 10^−3^, very close to the value for the anatomically accurate model, indicating that the rates of clearance in these two models are quite similar.

Contours for the remaining materials simulated resembled those for sucrose in the quasi steady-state phase, since they also could only leave by the anterior pathway. These concentration contour plots are quite similar to the contours measured experimentally by Araie and Maurice (57) after injection of fluorescein or fluorescently labeled dextrans in the rabbit vitreous. Similar profiles were also demonstrated previously (6).

**Fig. 15:**
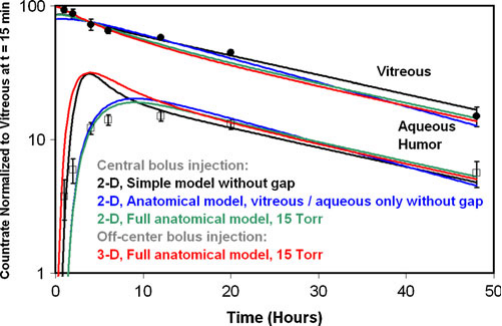
Simulation of intravitreal injection of ^14^C-sucrose in the rabbit. Data from reference (56). Simulation results from various geometric models as indicated.

A more quantitative assessment of the accuracy of these simulations in predicting the rate of clearance of injected material is shown in Fig. 15, which compares the simulation of intravitreal injection of ^14^C-sucrose in four models with experimental data (56). The simple axisymmetric model with the 45° opening without the gap accurately predicted the rate of decline of the vitreous concentration, once an adjustable parameter was used to multiply the simulation results (note that Bito *et al*. did not provide information that would have allowed for an absolute accounting for all injected radioactivity). Using this same adjustable parameter, the aqueous humor concentration was accurately predicted in the terminal phase after 12 h, although the simulations overshot the initial concentrations in the aqueous. The anatomically accurate models also matched the data in the vitreous and provided a better fit to the aqueous humor concentration at early time, devoid of overshoot, and matched the aqueous concentrations at later time.

The most likely reason the aqueous humor concentration profile predicted by the simplified model behaves differently at early time is because this model does not contain the lens, which serves as an obstruction around which the bolus must diffuse in order to reach the hyaloid boundary. If the injection is placed on the side of the vitreous, the early peak reappears in the simulation. (Note that this off-axis bolus injection simulation required use of the full 3-D geometry.) Thus we conclude that for this rapidly diffusing molecule which can only leave the vitreous by the anterior pathway, all the models provide accurate estimates for the rate of elimination from the vitreous and the aqueous/vitreous concentration ratio in the terminal phase.

**Fig. 16:**
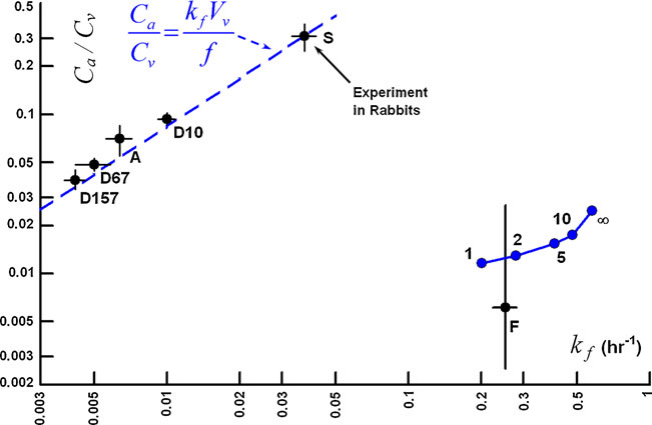
Log-log plot of aqueous/vitreous concentration ratio in the terminal phase *versus* vitreous elimination rate. F, Fluorescein, data from reference (58). S – sucrose, data from reference (56). D10, D67, D157, Dextrans (see Table VIII) – data from reference (59). A – bovine albumin, data from reference (60). Points in blue represent simulations of clearance of Fluorescein after IVT injection. Each point is labeled with the value of *P* used for the strength of the flux condition applied at the outer vitreous boundary in units of 10^−7^ M s^−1^.

Figure 16 shows experimental data for clearance behavior after IVT injection in the rabbit for the entire range of materials for which simulated were conducted in this study. This figure was constructed to be the equivalent to Figure 6-4 of reference (18). Data points sucrose, albumin, and the dextran polymers fall very close to the trend line predicted by Eq. 9, plotted as the dashed blue line. The data point for Fluorescein falls quite far from the trend line, with much faster elimination and much lower aqueous humor concentration. When an infinite sink was placed on all surfaces of the ciliary body and iris in contact with the aqueous humor and also at the rear of the vitreous, the aqueous humor concentration was predicted to be within the range observed experimentally, but the vitreous elimination rate was about three times too rapid. By applying a partial sink, equivalent to setting the value of *P* in the condition of Eq. 17 to a value somewhere between 1 and 2 × 10^−7^ m/s, the vitreous elimination rate comes into agreement with the observed value, and the aqueous concentration is still in the correct range.

**Fig. 17:**
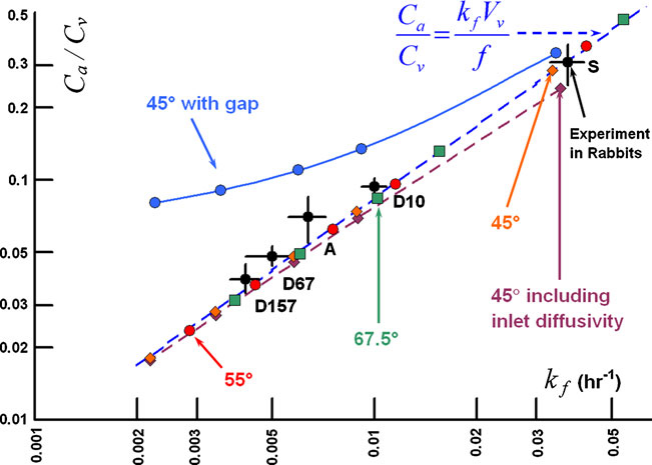
Clearance behavior in simulations of IVT injection of various materials in simplified axisymmetric models of Figs. 5 and 6. Simulations were conducted at diffusivities matching those in Table VIII for the materials sucrose (S), bovine albumin (A), and dextran having the molecular weights shown in the table. Dashed line shows the clearance behavior expected for materials that are cleared only by the anterior pathway (from Eq. 17). The angle of the hyaloid interface varied from 40° to 67.5° as shown. Diffusion through the inlet was disabled except for the single simulation shown. Only one model included the gap (Fig. 6); the remainder did not (Fig. 5).

Figure 17 shows the clearance behavior of the simplified axisymmetric models shown in Figs. 5 and 6 compared with the experimental data. Using this plot one can investigate the influence of adjusting the angle of placement of the fluid inlet and the presence or absence of the gap on the clearance behavior. Consider first the models without the gap (Fig. 5), for which the angle of the hyaloid membrane was varied from 45° to 67.5°. When diffusion through the inlet was disabled, simulation results fall directly on the line predicted by Eq. 9. The simulation point for sucrose predicted for the model with the 45° opening falls closest to the experimental value (which also falls directly on the equation line). The simulation point for this same model for D157 falls much lower than the experimental point, the predicted vitreous elimination rate being only about half the experimental rate.

When inlet diffusivity is enabled for the same model, the elimination rate increases and the aqueous concentration falls, since allowing material to diffuse through the fluid inlet amounts to applying a weak sink at the inlet surface. The influence is most noticeable for the more rapidly diffusing materials and is negligible for the slowest diffusers. For the remainder of the simulations presented in Fig. 17, inlet diffusivity was disabled. At the angle of exposure increases from 45° to 67.5°, the points slide up the curve, with the point predicted for D157 falling almost within the range covered by the experimental error — but the prediction for sucrose is also shifted to a value which is out of range of the experimental uncertainty. When the gap is included in the simplified axisymmetric model (Fig. 6), the simulation results fall on a curve above the line predicted by Eq. 9. Deviation from the line of Eq. 9 increases as diffusivity decreases.

**Fig. 18:**
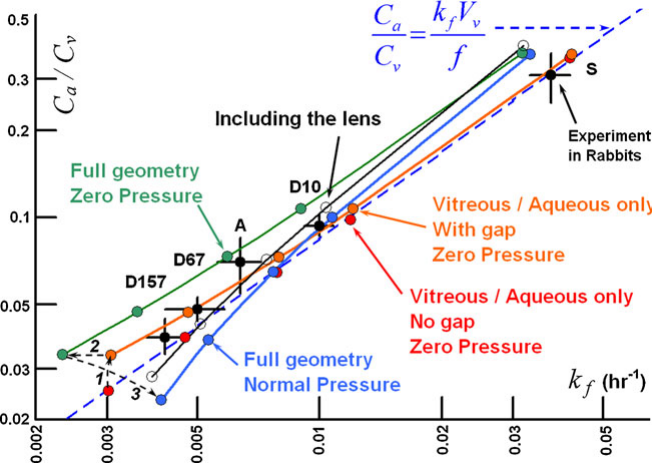
Clearance behavior in simulations of IVT injection of various materials in rabbit ocular models. All simulations conducted with inlet diffusivity disabled. Arrows ***1*** through ***3*** show how the clearance behavior changes as the model features are added. Arrow ***1***: effect of adding the gap. Arrow ***2***: effect of adding the outer shells at zero hydraulic pressure. Arrow ***3***: effect of hydraulic pressure of 15 Torr. Also shown in unfilled circles is the effect of adding the lens (also at hydraulic pressure).

Figure 18 shows the clearance behavior in the various anatomically accurate rabbit models. All simulations in this figure were conducted with inlet diffusivity disabled. The simulation results for the model comprised only of the vitreous and aqueous compartments without the gap fall directly on the line predicted by for Eq. 9, and are almost identical to the values produced from the axisymmetric model without a gap with a 55° exposure angle (see Fig. 17). These simulations severely underestimate the clearance rate for slowly diffusing materials. Including the gap increases the terminal elimination rate slightly and raises the aqueous concentration markedly, though not to the extent that occurred for the simple axisymmetric model which included the gap. This effect is represented by the dashed arrow labeled ***1***.

Next, including the outer shell tissues in the anatomically accurate model but conducting the simulation at a very low hydraulic pressure (0.1 Torr in excess of the episcleral venous pressure set at 10 Torr) reduces *k*_*f*_ by about 24%, further away from the experimental values especially at low diffusivity. But including the outer shell tissues does not the aqueous/vitreous concentration ratio. This effect is shown by arrow ***2***. Arrow ***3*** shows the impact of increasing intraocular pressure to the normotensive value of 15 Torr. The value of *k*_*f*_ increases and the aqueous/vitreous ratio falls. This brings the *k*_*f*_ values within the experimental error for most of the data, and causes the aqueous concentrations to be underpredicted for the more slowly diffusing materials. Including the lens raises the aqueous concentration without appreciably altering the elimination rate, bringing the simulations in closer to the experimental values.

**Fig. 19:**
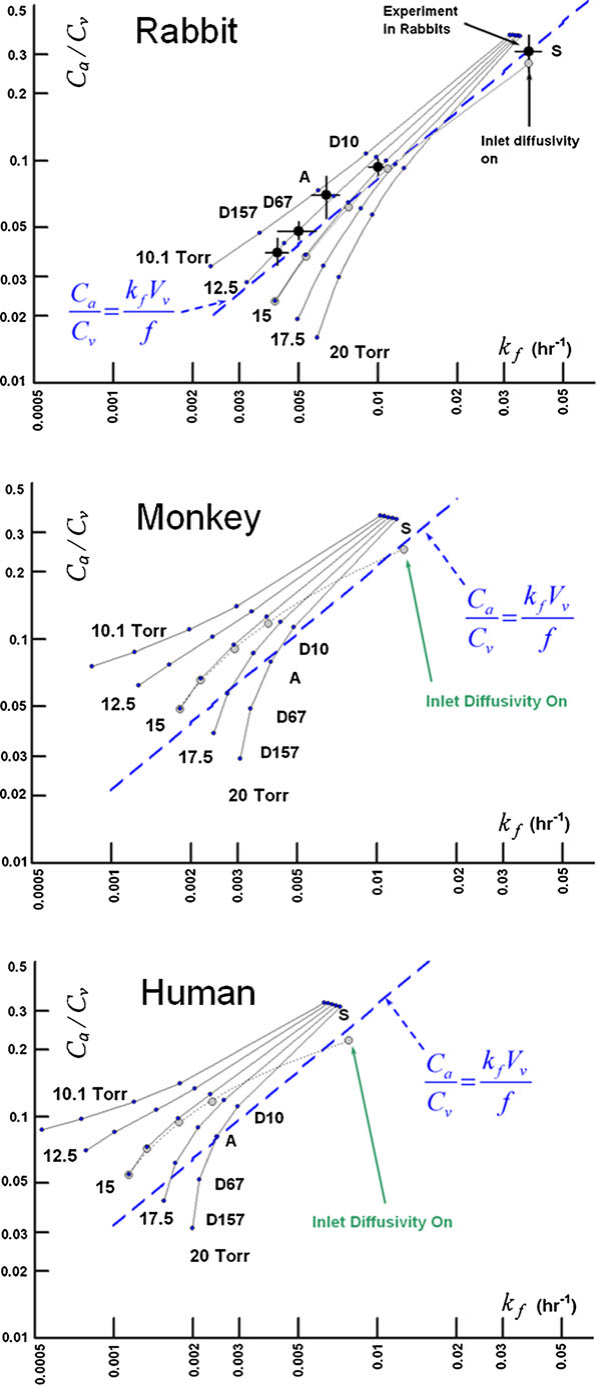
Influence of intraocular pressure on clearance after IVT injection in the three species model including all tissues. Inlet diffusivity was disabled except for the case of 15 Torr, for which the simulations were repeated with inlet diffusivity enabled. Blue trend lines were calculated from Eq. 9 using the vitreous volume and aqueous flow rate for each species model listed in Table II.

Figure 19 shows simulations for each of the species models conducted at several intraocular pressures for the series of injected materials. The trend line predicted by Eq. 9 is also presented, making the substitution for vitreous volume and aqueous flow rate appropriate for each species.

## DISCUSSION

The modeling approach used in this study explicitly represents the ocular anatomy and physiology in a geometric model. The rate of transfer between tissue compartments is governed by the way in which the geometry is constructed, the material properties assigned to the various tissue regions, and the boundary conditions for mass flow, pressure and concentration applied to various surfaces. Thus, the more accurately the anatomy and physiology is represented in the model, the more accurate will be the results obtained from the model. Wherever possible, the assignment of tissue properties should be anchored in independent experiments, to remove ambiguity and to reduce the number of variables to be adjusted by fitting to experimental data. In the current study the estimate for diffusivity for each of the various materials was anchored in physical measurements in aqueous solutions, and were most likely quite accurate estimates for their diffusivities in the aqueous and vitreous compartments, as corroborated by the excellent agreement in the predicted vitreous elimination rates. Assumptions were made regarding the diffusivities in other tissues based upon the known behavior of similar materials (50,51).

Reconstructing the exact shape of each compartment may not be as important as capturing some key features of the geometry which affect the transport between compartments. In this particular case there may not be much of an impact on the exact shape of the ellipsoidal structures, whether the eye is myopic or hyperopic, *etc*. But inaccuracy in the construction of the anterior compartment can be quite detrimental to the accuracy of the model in predicting clearance behavior after IVT injection. This was demonstrated in our previous study (6) which examined predictions in the rabbit geometries proposed by others (4,5).

In the current study, the effect of the small gap (the retrozonular space of Petit) which extends the aqueous humor chamber between the vitreous and ciliary body on the clearance of IVT injected materials was investigated, using experimental data available in the literature. A simplified axisymmetric model of the rabbit eye, which is little more than a caricature of the ocular anatomy, provided a means of investigating how the clearance of materials after IVT injection is affected by two aspects of the anatomy: 1) the unobstructed interface between the vitreous and aqueous compartments and 2) the narrow gap. The construction of the aqueous compartment was simplified to provide a rudimentary means of achieving a well-stirred condition, a requirement for the Maurice prediction of Eq. 9 (18) to be valid in the quasi steady-state.

The experimental data from injection of ^14^C-sucrose (Fig. 15) afforded a comparison with simulation results for a small, rapidly diffusing molecule whose transport in the vitreous is primarily driven by diffusion and is relatively unaffected by convection caused by hydraulic driven fluid flow. With the appropriate angle for the hyaloid opening, even the simplified axisymmetric models provided excellent predictions for the concentration — time profiles in both compartments after the quasi steady-state had been achieved. The anatomically accurate model provided simulation results that were in just as good agreement in the terminal phase, but provided a better agreement with the aqueous humor concentrations in the early phase. Unlike the simplified axisymmetric models, the anatomical model contained the lens, which served as an obstruction past which the bolus must diffuse, delaying its entry into the aqueous compartment.

When the gap is not included in either model, the simulations fall directly upon the trend line predicted by Eq. 9. The simulations for the anatomical model are almost identical to the values produced from the first simple axisymmetric model without a gap, despite the fact that hyaloid area is only half that for the simplified model. This indicates that the geometric influence on the rate of clearance from the vitreous is not simply a function of surface area of contact between the aqueous and vitreous compartments. Although the simulations without the gap fall on the same apparent curve for concentration ratio *versus* elimination rate as the experimental data, the predicted influence of diffusivity does not match. The clearance rate for slowly diffusing species is severely underestimated. This is because simulations without the gap were conducted at essentially zero intraocular pressure and thus there was no convection in the vitreous, which would have preferentially accelerated the rate of elimination for slowly diffusing materials.

Most unexpected are the results for the model with the gap, which reside on a curve quite different than predicted by Eq. 9, with slight increases in vitreous clearance rate but with marked increases in aqueous levels, especially with reduced diffusivity. Since the velocity of the fluid percolating through the vitreous is essentially zero for all of these models, this deviation must not have anything to do with hydraulic pressure-assisted convection within the vitreous and must be entirely a consequence of the altered geometry. The effect of adding the exterior shell tissues (represented by arrow ***2*** in Fig. 18) is to systematically shift the curve directly to the left, reducing the elimination rate by about the same proportion independent of diffusivity without changing the aqueous/vitreous ratio. This may reflect the influence of the slower diffusivity of material in the outer shells which slowly seeps material back into the vitreous during the terminal phase.

It is intraocular pressure that has the most significant impact on the clearance behavior for the slowly diffusing materials, apart from adding the gap, represented by arrow ***3*** in Fig. 18. Hydraulic pressure-induced convection in the anterior portion of the vitreous sweeps diffusing material away from the anterior and pushes it out of the vitreous through the outer shell tissues. This simultaneously reduces the aqueous concentration (since it reduces the concentration in the anterior vitreous, where the convection is the highest) and increases the elimination rate by adding another available pathway through the posterior tissues. These effects become more important as diffusivity decreases. The net effect is that the predictions for the rabbit eye at an intraocular pressure of 15 Torr fall somewhat close to the data. The elimination rate appears to be predicted more accurately than the aqueous humor concentration.

Just because the rabbit data falls on a trend line close to Eq. 9 doesn’t mean that the data for the other species will necessarily fall on their respective curves. This is evident in the normotensive simulations for the monkey and human species shown in Fig. 19, which tend to fall systematically above the trend line in each case. This suggests that the simple volumetric arguments usually used in the allometric scaling of experimental results from one species to another (61) may not always be appropriate. Another interesting implication of the curves of Fig. 19 is the potential effect of elevated intraocular pressure for slowly diffusing materials. It would seem that the vitreal elimination rate may eventually become independent of material diffusivity if the diffusivity becomes low enough.

One issue that cannot be resolved by the data is whether or not diffusion should be enabled through the fluid inlet. Although this setting exerts its greatest influence for rapidly diffusing materials, the change in clearance behavior predicted by either allowing or disabling diffusion through the fluid inlet is on the order of the experimental uncertainty. If the material diffusivity is low, then fortunately it does not matter whether or not this setting is applied, since it has little effect on the results.

Another issue that has not been carefully investigated is the influence of the thickness of the gap. The actual size of the gap is very difficult to estimate even from the highest resolution MRI images. The biologic variability in the size of the gap may also be important. Preliminary exploration of the influence of the size of the gap suggests that it may not be so much the thickness of the gap but the degree to which it extends toward the side of the eye that may be important in determining its impact on clearance. Also neglected is the potential impact of a pressure drop across the hyaloid membrane itself, since the default settings were applied to the porous-jump boundary, assigning a zero thickness value to the boundary.

The simple relationship between the aqueous/vitreous concentration ratio derived in Eq. 9, proposed more than 50 years ago (60), remains a very powerful tool for understanding the mechanisms which affect the disposition of IVT injected materials. For example, when the concentration ratio is extremely low and the clearance rate is extremely high, this suggests egress by the posterior pathway is important (see fluorescein (F) in Fig. 16). By applying a partial sink on the exterior vitreous boundary and infinite sinks on all surfaces for the iris and ciliary body in the anterior chamber, the clearance behavior of Fluorescein could be simulated within the range observed in *in vivo* experiments. We have recently found that some materials when injected into the eye have accelerated clearance (based on the prediction for a particular diffusivity) but do not have a reduced aqueous/vitreous concentration ratio. These materials appear to be cleared by a combination of the anterior pathway and some other mechanism operating in the bulk to clear the drug, such as degradation by an enzyme or complexation with some other agent. Preliminary examination of some of the data for Avastin seems to indicate that this may be happening (62,63).

This work can be improved in several ways. To simulate drug disposition in the lens, it would be necessary to include the lens in the geometry. Hydraulic and diffusion coefficients would need to be assigned. But the hydraulic resistance of the lens and its sheath is high, and diffusivity is very low. Thus if the main interest is to simulate the concentration in the vitreous, it should be safe to neglect the lens, as was done in this study.

To accurately estimate drug concentrations within the various tissues such as the retina, choroid, sclera, cornea iris and ciliary body, it is also required to obtain accurate values for hydraulic and diffusion coefficients for each of these tissues. In addition, it would be necessary to include boundary conditions to account for losses due to the influence of the vascular and lymphatic systems, possibly by applying a flux boundary condition similar to Eq. 17. These boundary conditions will also influence the clearance rate out from the vitreous, although their main influence will be confined to the exterior tissue layers. A better understanding of the anatomy and physiology of vascular flows in the choroid and episclera and of the lymphatic drainage mechanisms would possibly yield insights that could be incorporated into the model. The current model also does not offer any means for predicting drug concentration in the plasma compartment of the main circulatory system.

Another important feature to include in the model is chemical partitioning, which will cause the value of user-defined scalars to experience a discontinuity at the interface between two tissues having different partition coefficients for the drug. Methods have been developed previously to include this effect, which is more likely to be important for simulating the disposition of lipophilic drug (64). This method was limited to linear diffusion with constant partitioning, and may be improved upon to account for nonlinear (concentration-dependent) partitioning and diffusion (65). A similar feature to include in future methods is chemical binding (66).

The current simulations of advection use a very simple spherical shape for the initial bolus. In reality the process of intravitreal injection is quite complex, the result of the process of injection of one fluid into another with very different properties. Moreover, the intraocular pressure is elevated because the tissues of the outer sheath are elastic. Once the needle is withdrawn, a portion of the injected material may flow away from the region of injection by following along the path of the needle. Some of it may actually be expelled through the needle hole, but a portion may be drawn into the various layers between the tissues in the outer sheath, the layers between the retina and choroid or the choroid and sclera. If the site of injection is toward the anterior, it is possible that a portion of the bolus may actually be expelled immediately into the aqueous humor.

In these models only conventional outflow of aqueous humor through the trabecular meshwork was modeled. No attempt was made to incorporate uveoscleral outflow, in which a portion of the fluid passes through the ciliary muscle and into the suprachoroidal space (67,68). The current model does not represent the suprachoroidal space. The effect of not distinguishing between conventional and uveoscleral outflow pathways on the rate of clearance of material from the vitreous compartment is expected to be insignificant. Exactly where the fluid is cleared from the front of the eye is immaterial for predicting the concentration and clearance profiles of the vitreous. Whereas a small portion of the material cleared from the vitreous may be transported through the suprachoroidal space toward the back of the eye, its re-entry into the vitreous would be greatly hindered by the slow diffusivity in the sclera compared to the vitreous. The influence of this effect would be to very slightly reduce the clearance rate. This effect might become important when attempting to make accurate predictions of drug concentrations in the iris and ciliary body.

Another feature that may improve the predictions of aqueous humor concentrations is thermal convection (2,69). It is not likely that this effect will have a significant impact on the clearance rate from the vitreous. The lens is also likely to have an important impact. Shortly after injection, the lens would be loaded with diffusing material, which would act as a depot releasing slowly at later time. A proper treatment of the diffusion of material in the lens would require accounting for anisotropic diffusion effects (70).

The utility of the models for monkey and human will need to be tested by *in vivo* experimentation. Models for other species may be called for as they become utilized in the development of various drug therapies. The influence of the dimensions of gap and the accuracy of other aspects of the anatomical models warrants additional investigation.

## CONCLUSIONS

- With careful construction of the ocular anatomy and assignment of appropriate material properties and boundary conditions, this modeling strategy can accurately simulate the clearance of IVT injected materials having a range of diffusivity.
- Predictions of transport of slowly diffusing materials are more sensitive to the geometric structure of the ocular models because they are more prone to respond to the weak hydraulic flows in the vitreous.
- The simple linear relationship between the rate of egress of material from the vitreous and the mean concentration ratio between the aqueous humor and vitreous compartments, derived more than 60 years ago (60), continues to be a useful tool for interpretation of the disposition of IVT injected materials.
- The retrozonular space of Petit exerts an unexpected influence on the egress of slowly diffusing molecules, slightly increasing the vitreous clearance rate and markedly raising the aqueous/vitreous concentration ratio. This effect becomes more important as the diffusivity is reduced, and is also mediated by intraocular pressure.
- This method offers an alternative means for scaling experimental data from one species to another that may be more appropriate than other simple approaches based entirely upon scaling of compartment volumes.

## ABBREVIATIONS

*C*: concentration of diffusant
*C*_*a*_: average concentration of diffusant in the aqueous humor
*C*_*v*_: average concentration of diffusant in the vitreous
*D*: diffusion coefficient
*f*: aqueous humor flow rate
*H*: fluid viscosity
*k*_*B*_: Boltzmann constant
*k*_*f*_: fraction of material lost from the vitreous per unit time
*MW*: molecular weight
*ρ*: fluid density
*P*: permeability through the retina
*p*: pressure
*r*_*H*_: hydrodynamic radius
*T*: absolute temperature (°K)
*t*: time
$$ \overrightarrow v $$: velocity vector
*V*_*v*_: volume of the vitreous compartment
